# A case-control study on association of proteasome subunit beta 8 (*PSMB8*) and transporter associated with antigen processing 1 (*TAP1*) polymorphisms and their transcript levels in vitiligo from Gujarat

**DOI:** 10.1371/journal.pone.0180958

**Published:** 2017-07-10

**Authors:** Shahnawaz D. Jadeja, Mohmmad Shoab Mansuri, Mala Singh, Mitesh Dwivedi, Naresh C. Laddha, Rasheedunnisa Begum

**Affiliations:** Department of Biochemistry, Faculty of Science, The Maharaja Sayajirao University of Baroda, Vadodara, Gujarat, India; University of Alabama at Birmingham, UNITED STATES

## Abstract

**Background:**

Autoimmunity has been implicated in the destruction of melanocytes from vitiligo skin. Major histocompatibility complex (MHC) class-II linked genes proteasome subunit beta 8 *(PSMB8)* and transporter associated with antigen processing 1 *(TAP1)*, involved in antigen processing and presentation have been reported to be associated with several autoimmune diseases including vitiligo.

**Objectives:**

To explore *PSMB8* rs2071464 and *TAP1* rs1135216 single nucleotide polymorphisms and to estimate the expression of *PSMB8* and *TAP1* in patients with vitiligo and unaffected controls from Gujarat.

**Methods:**

*PSMB8* rs2071464 polymorphism was genotyped using polymerase chain reaction- restriction fragment length polymorphism (PCR-RFLP) and *TAP1* rs1135216 polymorphism was genotyped by amplification refractory mutation system-polymerase chain reaction (ARMS-PCR) in 378 patients with vitiligo and 509 controls. Transcript levels of *PSMB8* and *TAP1* were measured in the PBMCs of 91 patients and 96 controls by using qPCR. Protein levels of PSMB8 were also determined by Western blot analysis.

**Results:**

The frequency of ‘TT’ genotype of *PSMB8* polymorphism was significantly lowered in patients with generalized and active vitiligo (*p =* 0.019 and *p =* 0.005) as compared to controls suggesting its association with the activity of the disease. However, *TAP1* polymorphism was not associated with vitiligo susceptibility. A significant decrease in expression of *PSMB8* at both transcript level (*p* = 0.002) as well as protein level (*p* = 0.0460) was observed in vitiligo patients as compared to controls. No significant difference was observed between patients and controls for *TAP1* transcripts (*p* = 0.553). Interestingly, individuals with the susceptible CC genotype of *PSMB8* polymorphism showed significantly reduced *PSMB8* transcript level as compared to that of CT and TT genotypes (*p* = 0.009 and *p* = 0.003 respectively).

**Conclusions:**

*PSMB8* rs2071464 was associated with generalized and active vitiligo from Gujarat whereas *TAP1* rs1135216 showed no association. The down-regulation of *PSMB8* in patients with risk genotype ‘CC’ advocates the vital role of *PSMB8* in the autoimmune basis of vitiligo.

## Introduction

Vitiligo, a cosmetic disfigurement disorder, may lead to psychological and social stigma, particularly in people with dark and intermediate skin tones. It is characterized by circumscribed milky white patches on the skin affecting about 0.06–2.28% of the world population [1]. Based on a few dermatological outpatient records, the prevalence of vitiligo is found to be 0.5 to 2.5% in India [2], wherein Gujarat and Rajasthan states have the high prevalence i.e. ~8.8% [3]. The exact etiopathology of vitiligo is not defined, however, based on extensive studies various theories such as oxidative stress, autoimmunity, and neurochemical hypothesis have been proposed to explain the underlying pathomechanisms [4–7]. Autoimmunity has been strongly involved in the development of disease, as 30% of vitiligo cases are affected with at least one of the concomitant autoimmune disorders [5,6]. Several studies including ours have identified critical role of CD8^+^ cytotoxic T cells in melanocyte destruction [8,9]. Generation of antigenic peptides and their transport across the membrane of the endoplasmic reticulum for assembly with major histocompatibility complex (MHC) class I molecules are essential steps in antigen presentation to cytotoxic T lymphocytes [10]. Genes within MHC class II loci along with genes involved in antigen processing and presentation i.e., proteasome subunit beta 8 *(PSMB8)* and transporter associated with antigen processing 1 *(TAP1)* have been reported to be associated with several autoimmune diseases including vitiligo [11–19]. The *PSMB8*, often referred as *LMP7* encodes interferon (IFN)-γ inducible subunit of immune proteasome i.e., β5i involved in degradation of ubiquitinated intracellular proteins into peptides that are especially suited for presentation by MHC class I molecules. Whereas, *TAP1* encode a subunit of an IFN-γ inducible heterodimer which binds with peptides cleaved by the proteasome and transports them to be loaded into nascent MHC class I molecules for presentation to CD8^+^ T cells [20,21].

The genome-wide association study (GWAS) on generalized vitiligo revealed that the association of *TAP1-PSMB8* seems to derive from linkage disequilibrium with major primary signals in the MHC class I and class II regions [17]. Out of 8 different single nucleotide polymorphisms (SNPs) of *PSMB* and *TAP* gene region studied, *PSMB8* intron 6 G/T and *TAP1* exon 10 A/G were found to be significantly associated with vitiligo in the Western population [14]. Another study showed significant association of *TAP1* exon 10 A/G polymorphism with vitiligo in Saudi population but not for *PSMB8* intron 6 G/T polymorphism [22]. The nature of the genetic association may vary according to different ethnic backgrounds. However, despite having high prevalence of vitiligo in Gujarat, there are no reports of *PSMB8* and *TAP1* polymorphisms so far. Hence, the present study aims, (i) to investigate the association of *PSMB8* intron 6 (rs2071464) and *TAP1* exon 10 (rs1135216) polymorphisms and (ii) to estimate transcript levels of *PSMB8* and *TAP1* using a case-control approach.

## Materials and methods

### Study subjects

We report a case-control study including 509 ethnically age and gender matched controls and 378 patients with vitiligo from Gujarat. Unaffected individuals of age between 5 to 60 years were recruited in the study. None of the unaffected individuals had any evidence of vitiligo and any other disease. Patients with vitiligo who referred to S.S.G. Hospital at Vadodara, Gujarat, India were recruited in the study. The inclusion criteria followed were: outpatients of age between 5 to 60 years and both the parents should be Gujarati by birth. Patients with other diseases and those unwilling to participate in the study were excluded. The diagnosis of vitiligo by dermatologists was clinically based on characteristic skin depigmentation with typical localization and white color lesions on the skin, under Woods lamp. Generalized or non-segmental vitiligo (GV) was characterized by depigmented patches varying in size from a few to several centimeters in diameter, involving one or both sides of the body with a tendency towards symmetrical distribution [23]. Whereas localized or segmental vitiligo (LV) typically has a rapidly progressive but limited course, depigmentation spreads within the segment during a period of 6–24 months and then stops; further extension is rare [23]. Following clinical criteria to proposed by Falabella *et al*., [24] and discussed in the Vitiligo Global Issues Consensus Conference 2012 [23], were used for characterizing stable vitiligo (SV): (i) lack of progression of old lesions within the past 2 years; (ii) no new lesions developing within the same period. Active vitiligo (AV) was defined as the appearance of new lesions and spreading of existing lesions observed during past two-year duration. The importance of the study was explained to all participants and written consent was obtained. Informed consent in written was obtained from the next of kin, caretakers, or guardians on behalf of the minors/children enrolled in the study. The study plan and consent forms were approved by the Institutional ethical committee for human research (IECHR), Faculty of Science, The Maharaja Sayajirao University of Baroda, Vadodara, Gujarat, India (FS/IECHR/BC/RB/1). Demographic characteristics of the patients are provided in Supporting information as ‘S1 Table’.

### Genomic DNA extraction

Genomic DNA was extracted from PBMCs using ‘QIAamp^TM^ DNA Blood Kit’ (QIAGEN Inc., Valencia, CA 91355, USA) according to the manufacturer’s instructions. After extraction, concentration and purity of DNA were estimated spectrophotometrically, quality of DNA was also determined on 0.8% agarose gel electrophoresis and DNA was stored at -20°C until further analyses.

### Genotyping of *PSMB8* rs2071464 polymorphism

Polymerase chain reaction- Restriction Fragment Length Polymorphism (PCR-RFLP) technique was used to genotype *PSMB8* rs2071464 polymorphism. The primers used for polymerase chain reaction are mentioned in S2 Table. The reaction mixture of the total volume of 20 μL included 3 μL (100ng) of genomic DNA, 11 μL nuclease-free H_2_O, 2.0 μL 10x PCR buffer, 2 μL 2 mM dNTPs (Genei^TM^, Bangalore, India), 1 μL of 10 pM corresponding forward and reverse primers (Eurofins^TM^, India), and 0.3 μL (3 U/μL) Taq Polymerase (Genei^TM^, Bangalore, India). Amplification was performed Eppendorf Mastercycler Gradient Thermocycler (Eppendorf^TM^, Germany) according to the protocol: 95°C for 10 minutes followed by 45 cycles of 95°C for 30 seconds, 58°C for 30 seconds and 72°C for 30 seconds, and 72°C for 10 minutes. The amplified products were checked by electrophoresis on a 2.0% agarose gel stained with ethidium bromide. Restriction enzyme was used for digesting the PCR product (S2 Table). 15 μL of the amplified products were digested with 1U of *Hha* I (Fermentas^TM^, Thermo Scientific, Waltham, MA) in a total reaction volume of 20μL as per the manufacturer’s instruction. The digestion products were resolved with 50 bp DNA ladder (Novagen^TM^, Perfect DNA ladder) on 3.5% agarose gel stained with ethidium bromide and visualized under E-Gel Imager (Life Technologies^TM^, Carlsbad, CA). Representative gel image is shown in S1 Fig. More than 10% of the samples were randomly selected for confirmation and the results were 100% concordant (analysis of the chosen samples was repeated by two researchers independently). Six samples of each genotype were also confirmed by sequencing (S2 Fig) using carefully designed primers (S3 Table).

### Genotyping of *TAP1* rs1135216 polymorphism

*TAP1* rs1135216 polymorphism was genotyped using amplification refractory mutation system-polymerase chain reaction (ARMS-PCR) method. DNA was amplified in two different PCR reactions with a generic antisense primer and one of the two allele-specific sense primers (S2 Table). To assess the success of PCR amplification in both the reactions, an internal control of 407 bp was amplified using a pair of primers designed from the nucleotide sequence of the human growth hormone (*HGH*) (S2 Table). The reaction mixture of the total volume of 15 μL included 3 μL (100 ng) of genomic DNA, 4.7 μL nuclease-free H_2_O, 1.5 μL 10x PCR buffer, 1.5 μL 2mM dNTPs (Genei^TM^, Bangalore, India), 1 μL of 10 pM allele- specific and common primers (Eurofins^TM^, India), 1 μL of 10 pM control primers (*HGH*), and 0.3 μL (3U/μL) Taq Polymerase (Genei^TM^, Bangalore, India). Amplification was performed using a Mastercycler Gradient PCR (Eppendorf^TM^, Germany) according to the protocol: 95°C for 10 minutes followed by 45 cycles of 95°C for 30 seconds, 61°C for 30 seconds, and 72°C for 30 seconds, and 72°C for 10 min. The PCR products were resolved on 3.5% agarose gel stained with ethidium bromide along with 50bp DNA ladder (Novagen^TM^, Perfect DNA ladder) and visualized under E-Gel Imager (Life Technologies^TM^, Carlsbad, CA). Two amplicons were available for each sample (one each specific for A or G allele). Representative gel image is shown in S1 Fig. More than 10% of the samples were randomly selected for confirmation and the results were 100% concordant (analysis of the chosen samples was repeated by two researchers independently). Six samples of each genotype were also confirmed by sequencing (S3 Fig) using carefully designed primers (S3 Table).

### Estimation of *PSMB8* and *TAP1* transcript levels

#### RNA extraction and cDNA synthesis

Total RNA from PBMCs was isolated and purified using the Ribopure-blood Kit (Ambion^TM^ Inc., Austin, TX, U.S.A.) following the manufacturer’s protocol. RNA integrity was verified by 1.5% agarose gel electrophoresis, RNA yield and purity was determined spectrophotometrically at 260/280 nm. RNA was treated with DNase I (Ambion^TM^ inc. Texas, USA) before cDNA synthesis to avoid DNA contamination. cDNA synthesis was performed using 1 μg of total RNA by RevertAid First Strand cDNA Synthesis Kit (Fermentas^TM^, Vilnius, Lithuania) according to the manufacturer’s instructions in Eppendorf Mastercycler Gradient Thermocycler (Eppendorf^TM^, Germany).

#### Quantitative realtime PCR (qPCR)

The expression of *PSMB8*, *TAP1* and Glyceraldehyde 3-phosphate dehydrogenase (*GAPDH*) transcripts were measured by qPCR using gene specific primers (Eurofins^TM^, Bangalore, India) as shown in S4 Table. Expression of the *GAPDH* gene was used as a reference. qPCR was performed in duplicates in 20 μl volume using LightCycler^®^ 480 SYBR Green I Master (Roche^TM^ Diagnostics GmbH, Mannheim, Germany) following the manufacturer’s instructions. The thermal cycling conditions included an initial activation step at 95°C for 10 min, followed by 45 cycles of denaturation, annealing, and extension (95°C for 10 sec, 65°C for 15 sec, 72°C for 20 sec). The fluorescence data collection was performed during the extension step. At the end of the amplification phase, a melt curve analysis was carried out to check the specificity of the products formed. The PCR cycle at which PCR amplification begins its exponential phase and product fluorescence intensity finally rises above the background and becomes visible was considered as the crossing point-PCR-cycle (C_P_) or cycle threshold (C_T_). The ΔC_P_ value was determined as the difference between the cycle threshold of target genes (*PSMB8/TAP1*) and reference gene (*GAPDH*). The difference between the two ΔC_P_ values (ΔC_P_ Controls and ΔC_P_ patients) was considered as ΔΔC_P_ to obtain the value of fold expression (2^-ΔΔCp^).

### Estimation of PSMB8 protein expression

#### Western blot analysis

Five ml blood was drawn from healthy controls and patients with active GV and collected in EDTA vials. Red blood cells were lysed with RBC lysis buffer (0.17 M Tris/ 0.16 M NH_4_Cl pH 7.2) and the remaining leukocytes were washed in PBS, and lysed in lysis buffer (1 mM EDTA, 50 mM Tris-HCl pH 7.5, 70 mM NaCl, 1% Triton, 50 mM NaF) containing 1x proteinase inhibitors (Sigma, Bangalore, India). Protein concentration was determined by Bradford assay (HiMedia Laboratories, India) and 20μg protein was loaded on 12% SDS-PAGE along with Precision Plus Protein™ Dual Color Standards (Bio-Rad, Germany). Protein was electro-blotted on PVDF membrane at 100 V for 1.5 hrs. Following the transfer, the membrane was blocked with 5% blocking buffer (5% BSA and 0.1% Tween-20 in PBS) for 1 hr at room temperature. The membrane was incubated overnight with primary antibody against LMP7/PSMB8 (ab58094). After incubation the membrane was washed four times with PBS-T (PBS containing 0.1% Tween 20) for 15 min. and incubated with a secondary anti-mouse antibody (Bangalore Genei, India) at room temperature for 1 hr. The membrane was similarly washed four times with PBS-T and protein bands on the membrane were then visualized by using Bio-Rad Clarity™ western ECL substrate (Bio-Rad, Germany) and signal was scanned using the Chemidoc™ Touch Gel Imaging System (Bio-Rad, Germany). Intensities of target proteins were normalized with that of total protein loading by staining the membrane with Ponceau. Densitometric analysis of the protein bands was calculated by ImageJ software.

### Statistical analyses

Hardy-Weinberg equilibrium (HWE) was evaluated for both SNPs in patients and controls by comparing the observed and expected frequencies of the genotypes using chi-square analysis. Distribution of the genotypes and allele frequencies of polymorphisms in different groups were compared using chi-square test with 2×2 contingency tables. Major genotype/allele was used as a reference. Multiple comparisons were controlled by the Bonferroni’s method. Odds ratio (OR) with 95% confidence interval (CI) for disease susceptibility was also calculated. Haplotype and LD analysis were carried out using http://analysis.bio-x.cn/myAnalysis.php [25]. For analyses of the transcript and protein levels unpaired t-test and one-way ANOVA were applied. Tukey’s multiple correction was applied for multiple testing and the p-values were adjusted. All the statistical tests were carried out using Prism 6 software (Graph Pad Software, USA).

### Bioinformatics analysis

*In silico* prediction tools HaploReg v4.1 [26] and Regulome DB [27] were employed to predict the functional impact of non-coding polymorphism. *In silico* prediction tools SIFT [28], PANTHER [29], I-MUTANT SUITE [30], POLYPHEN [31], MUPRO [32] were employed to predict the impact on the protein due to single amino acid variation. SNPs and GO [33] predicts the variation effect which might terminate into a disease like a trait. The details have been described in ‘Supporting Information’ file (S1 Text).

## Results

### *PSMB8* rs2071464 polymorphism in vitiligo

Genotyping of *PSMB8* intron 6 rs2071464 SNP by PCR-RFLP using *Hha* I and subsequent sequencing results revealed that there is C>T nucleotide change instead of previously reported G>T change, which falls in the *Hha* I recognition/restriction site and was imputed to *PSMB8* rs2071464 SNP [12,19,34,35]. The observed genotype frequencies of *PSMB8* rs2071464 SNP among the controls were in accordance (*p* = 0.071) whereas, genotype frequencies among the patients were deviated (*p* = 0.001) from HWE. When ‘C’ allele and CC genotype were used as reference group, the frequencies of the variant ‘T’ allele and homozygous ‘TT’ genotype were significantly lower in patients with vitiligo as compared to controls (49% vs. 54%, *p* = 0.031; 19% vs. 27%, *p* = 0.026 respectively) but it did not remain significant after Bonferroni’s correction. The protective role of ‘TT’ genotype in patients was suggested by OR = 0.629 (95% CI = 0.41–0.94). OR suggests that the minor allele ‘T’ might have the protective role in the disease pathogenesis (Table 1). Analysis based on types of vitiligo revealed significantly lower frequency of ‘TT’ genotype (18% vs. 27%, *p* = 0.019) and ‘T’ allele (48% vs. 54%, *p* = 0.024) in patients with GV as compared to controls. No significant difference in genotype and allele frequencies between patients with LV in comparison to patients with GV or controls (Table 2). Interestingly, a similar trend was observed upon analysis based on the activity of the disease (Table 3). Predominantly increased frequency of the risk genotype ‘CC’ (24% vs.19%) and allele ‘C’ (53% vs. 46%) was observed in patients with AV as compared to controls. The frequency of the protective genotype ‘TT’ (18% vs. 27%, *p* = 0.005) and allele ‘T’ (47% vs. 54%, *p* = 0.007) was significantly lowered in comparison to controls. However, no significant difference in allele and genotype frequencies was observed between patients with AV and SV.

**Table 1:** Association of *PSMB8* and *TAP1* polymorphisms in patients with vitiligo from Gujarat.

SNP	Genotype/Allele	Patients n = 378 (Freq.)	Controls n = 509 (Freq.)	*p* for Association	Odds ratio	CI (95%)	*p* for HWE
***PSMB8* rs2071464**	Genotype						0.071 (C) 0.001 (P)
	CC	82 (0.22)	97 (0.19)	R	1	-	
	CT	222 (0.59)	273 (0.54)	0.825^a^	0.961^a^	0.68–1.35^a^	
	TT	74 (0.19)	139 (0.27)	0.026^a^	0.629^a^	0.41–0.94^a^	
	Allele						
	C	386 (0.51)	467 (0.46)	R	1	-	
	T	370 (0.49)	551 (0.54)	0.031^b^	0.812^b^	0.67–0.98^b^	
***TAP1* rs1135216**	Genotype						0.663 (C) 0.167 (P)
	AA	263 (0.70)	341 (0.67)	R	1	-	
	AG	100 (0.26)	153 (0.30)	0.278^a^	0.847^a^	0.63–1.14^a^	
	GG	15 (0.04)	15 (0.04)	0.487^a^	1.297^a^	0.62–2.70^a^	
	Allele						
	A	626 (0.83)	835 (0.82)	R	1	-	
	G	130 (0.17)	183 (0.18)	0.670^b^	0.950^b^	0.74–1.21^b^	

‘n’ represents number of Patients/ Controls,

‘R’ represents reference group,

HWE refers to Hardy-Weinberg Equilibrium,

CI refers to Confidence Interval, Odds ratio is based on allele frequency distribution.

(P) refers to Patients and (C) refers to Controls,

^a^Patients vs. Controls (genotype) using chi-squared test with 2 × 2 contingency table,

^b^Patients vs. Controls (allele) using chi-squared test with 2 × 2 contingency table,

Statistical significance was considered at p value ≤ 0.025 due to Bonferroni’s correction.

**Table 2:** Association of *PSMB8* and *TAP1* polymorphisms in patients with generalized and localized vitiligo from Gujarat.

SNP	Genotype /Allele	Generalized Vitiligo n = 292 (Freq.)	Localized Vitiligo n = 86 (Freq.)	Controls n = 509 (Freq.)	*p* for Association	Odds ratio	CI (95%)
***PSMB8* rs2071464**	Genotype						
	CC	64 (0.22)	18 (0.21)	97 (0.19)	R	1	-
	CT	174 (0.60)	48 (0.56)	273 (0.54)	0.951^a^	1.020^a^	0.55–1.88^a^
					0.854^b^	0.966^b^	0.67–1.40^b^
					0.858^c^	0.947^c^	0.52–1.70^c^
	TT	54 (0.18)	20 (0.23)	139 (0.27)	0.461^a^	0.759^a^	0.36–1.58^a^
					0.019^b^	0.588^b^	0.37–0.92^b^
					0.468^c^	0.775^c^	0.39–1.54^c^
	Allele						
	C	302 (0.52)	84 (0.49)	467 (0.46)	R	1	-
	T	282 (0.48)	88 (0.51)	551 (0.54)	0.507^a^	0.891^a^	0.63–1.25^a^
					0.024^b^	0.791^b^	0.64–0.97^b^
					0.471^c^	0.887^c^	0.64–1.23^c^
***TAP1* rs1135216**	Genotype						
	AA	203 (0.69)	60 (0.70)	341 (0.67)	R	1	-
	AG	78 (0.27)	22 (0.26)	153 (0.30)	0.868^a^	1.048^a^	0.60–1.82^a^
					0.347^b^	0.856^b^	0.62–1.18^b^
					0.450^c^	0.817^c^	0.48–1.38^c^
	GG	11 (0.04)	4 (0.04)	15 (0.04)	0.730^a^	0.812^a^	0.25–2.64^a^
					0.608^b^	1.232^b^	0.55–2.73^b^
					0.470^c^	1.516^c^	0.49–4.72^c^
	Allele						
	A	484 (0.83)	142 (0.88)	835 (0.82)	R	1	-
	G	100 (0.17)	30 (0.12)	183 (0.18)	0.922^a^	0.978^a^	0.62–1.53^a^
					0.666^b^	0.942^b^	0.72–1.23^b^
					0.866^c^	0.964^c^	0.63–1.47^c^

‘n’ represents number of Patients/ Controls,

‘R’ represents reference group,

CI refers to Confidence Interval, Odds ratio is based on allele frequency distribution.

^a^Generalized vitiligo vs. Localized vitiligo,

^b^Generalized vitiligo vs. Controls,

^c^Localized vitiligo vs. Controls,

Statistical significance was considered at p < 0.025 due to Bonferroni’s correction.

**Table 3:** Association of *PSMB8* and *TAP1* polymorphisms in patients with active and stable vitiligo from Gujarat.

SNP	Genotype / Allele	Active Vitiligo n = 305 (Freq.)	Stable Vitiligo n = 73 (Freq.)	Controls n = 509 (Freq.)	*p* for Association	Odds ratio	CI (95%)
***PSMB8* rs2071464**	Genotype						
	CC	72 (0.24)	10 (0.16)	97 (0.19)	R	1	-
	CT	178 (0.58)	44 (0.60)	273 (0.54)	0.123^a^	0.562^a^	0.27–1.18^a^
					0.478^b^	0.878^b^	0.61–1.26^b^
					0.224^c^	1.563^c^	0.76–3.23^c^
	TT	55 (0.18)	19 (0.24)	139 (0.27)	0.031^a^	0.402^a^	0.17–0.93^a^
					0.005^b^	0.533^b^	0.34–0.82^a^
					0.493^c^	1.326^c^	0.59–2.98^c^
	Allele						
	C	322 (0.53)	64 (0.44)	467 (0.46)	R	1	-
	T	288 (0.47)	82 (0.56)	551 (0.54)	0.052 ^a^	0.698^a^	0.48–1.00^a^
					0.007^b^	0.758^b^	0.62–0.93^b^
					0.644^c^	1.086^c^	0.76–1.54^c^
***TAP1* rs1135216**	Genotype						
	AA	205 (0.67)	58 (0.80)	341 (0.67)	R	1	-
	AG	86 (0.28)	14 (0.19)	153 (0.30)	0.086^a^	1.738^a^	0.92–3.28^a^
					0.677^b^	0.935^b^	0.68–1.28^b^
					0.045^c^	0.538^c^	0.29–0.99^c^
	GG	14 (0.05)	01 (0.01)	15 (0.04)	0.156^a^	3.961^a^	0.51–30.77^a^
					0.246^b^	1.553^b^	0.73–3.28^b^
					0.352^c^	0.392^c^	0.05–3.02^c^
	Allele						
	A	496 (0.81)	130 (0.89)	835 (0.82)	R	1	-
	G	114 (0.19)	16 (0.11)	183 (0.18)	0.026^a^	1.867^a^	1.07–3.26^a^
					0.719^b^	1.049^b^	0.81–1.36^b^
					0.035^c^	0.561^c^	0.32–0.96^c^

‘n’ represents number of Patients/ Controls,

‘R’ represents reference group,

CI refers to Confidence Interval, Odds ratio is based on allele frequency distribution.

^a^Active Vitiligo vs. Stable Vitiligo,

^b^Active Vitiligo vs. Controls,

^c^Stable Vitiligo vs. Controls,

Statistical significance was considered at p < 0.025 due to Bonferroni’s correction.

### *TAP1* rs1135216 polymorphism in Vitiligo

Both, control and patient groups were following HWE (*p* = 0.663 and *p* = 0.167 respectively; Table 1). Major allele ‘A’ and ‘AA’ genotype were considered as the reference. The allele and genotype frequencies were not significantly different in patients and control (Table 1). *TAP1* SNP when analyzed based on the type of vitiligo, no significant difference in genotype and allele frequencies was observed between patients with GV and LV with respect to unaffected controls (Table 2). Analysis based on the activity of the disease also showed no significant difference among the genotypes as well as allele frequencies (Table 3).

### Linkage disequilibrium and haplotype analyses

LD analysis revealed that two polymorphisms investigated i.e., *PSMB8* rs2071464 and *TAP1* rs1135216 were in low LD association (D’ = 0.432, r^2^ = 0.044). Haplotype evaluation of the two polymorphic sites was performed and the estimated frequencies of the haplotypes were not significantly different between patients and controls (global *p* = 0.278; Table 4).

**Table 4:** Distribution of haplotypes frequencies for *PSMB8* (C/T) and *TAP1* (A/G) polymorphisms in vitiligo patients and controls.

Haplotype [*PSMB8* (C/T): *TAP1* (A/G)]	Patients (Freq) n = 742	Control (Freq) n = 974	*p* for association	*p* (Global)	Odds Ratio [95%CI]
C A	222 (0.38)	194 (0.31)	0.058	0.278	1.26 [0.99~1.60]
C G	72 (0.12)	75 (0.27)	0.904		0.98 [0.69~1.38]
T A	262 (0.45)	296 (0.15)	0.092		0.82 [0.65~1.03]
T G	30 (0.05)	31 (0.27)	0.908		0.97 [0.58~1.62]

CI represents Confidence Interval,

(Frequency <0.03 in both control & case has been dropped and was ignored in analysis).

### *PSMB8* transcript and protein levels in vitiligo

**Fig 1:**
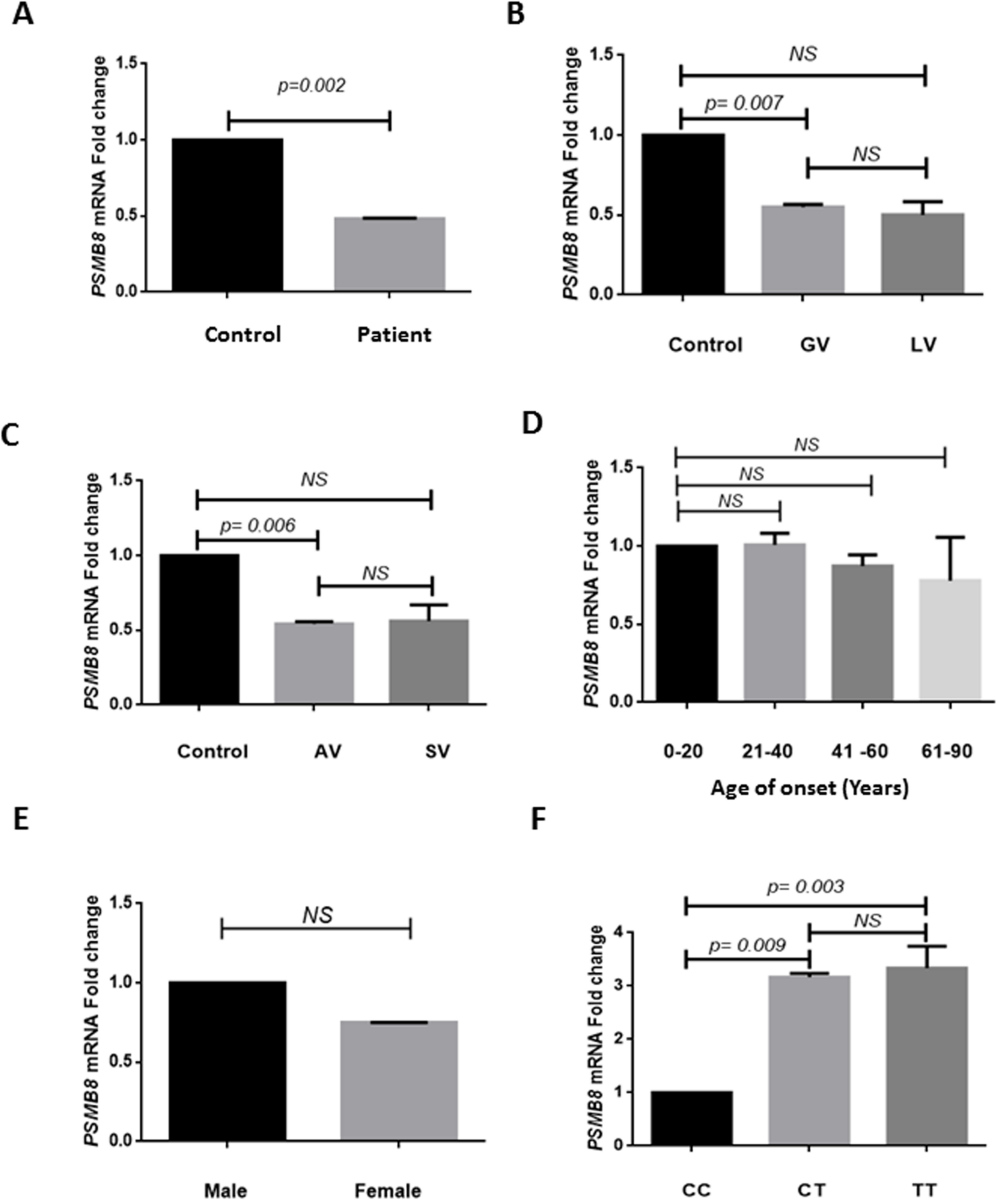
Relative gene expression of *PSMB8* in cases and controls. (A) Expression of *PSMB8* transcripts in 96 controls (52 male and 44 female), 91 patients with vitiligo (48 male and 43 female) was analyzed by applying unpaired t-test. Patients showed a significant decrease in transcript levels of *PSMB8* compared to controls (mean ΔCp ± SEM: 8.958±0.239 vs 10.01 ± 0.229; *p* = 0.002). Expression of *PSMB8* transcripts in patients against controls showed 0.52 -fold decrease as determined by the 2^-ΔΔCp^ method. (B) Expression of *PSMB8* transcripts in 96 controls and 72 patients with GV and 19 patients with LV was analyzed by using one-way ANOVA. Patients with GV showed significantly decreased *PSMB8* transcript levels as compared to controls (*p* = 0.007). However, there was no significant difference in *PSMB8* transcript levels between patients with GV and LV as well as in patients with LV as compared to controls (*p* = 0.975 and *p* = 0.090, respectively). (C) Expression of *PSMB8* transcripts in 96 controls and 69 patients with AV and 22 patients with SV was analyzed by using one-way ANOVA. Patients with AV showed significantly decreased *PSMB8* transcript levels as compared to controls (*p* = 0.006). However, there was no significant difference in *PSMB8* transcript levels between patients with AV and SV as well as in patients with SV as compared to controls (*p* = 0.999 and *p* = 0.112, respectively). (D) Expression of *PSMB8* transcripts with respect to different age of onset groups in 91 patients with vitiligo was analyzed by using one-way ANOVA. No significant difference in *PSMB8* transcript levels was observed in patients with respect to different age of onset groups. (E) Expression of *PSMB8* transcripts with respect to sex differences in 48 male and 43 female patients was analyzed by applying unpaired t-test. No significant difference was observed in both the groups (*p* = 0.396). (F) Expression of *PSMB8* transcripts with respect to the *PSMB8* rs2071464 SNP in 96 controls and 91 patients was analyzed by using one-way ANOVA. Individuals with the CC genotype showed decreased *PSMB8* transcripts when compared with CT and TT genotypes (*p* = 0.009 and *p* = 0.003, respectively). No significant difference in *PSMB8* transcripts levels was observed in individuals with the CT and TT genotypes (*p* = 0.448).

Analysis of *PSMB8* transcript levels revealed a significant decrease in expression of *PSMB8* transcripts in patients as compared to controls (*p* = 0.002; Fig 1A) after normalization with *GAPDH* expression. The 2^-ΔΔCp^ analysis showed approximately 0.52-fold decrease in the expression of *PSMB8* transcript levels in patients, as compared to controls (Fig 1A). Interestingly, analysis based on type and activity of the disease revealed that *PSMB8* transcript levels were significantly decreased in patients with GV as well as AV in comparison to controls (*p* = 0.007 and *p* = 0.006 respectively; Fig 1B and 1C), suggesting a role in the autoimmune basis of the disease. However, there was no significant difference in patients with LV and SV as compared to controls (*p* = 0.090 and *p* = 0.112 respectively; Fig 1B and 1C). Also, no significant difference in transcript levels was observed between GV vs LV and AV vs SV patients (Fig 1B and 1C). When expression of *PSMB8* transcripts was monitored in different age at onset groups of patients, no significant difference was observed in any of the age of onset groups i.e., 21–40, 41–60 and 61–80 years when compared with 1–20 years (Fig 1D). Gender-based analysis also showed no significant difference in *PSMB8* transcripts in both the groups (*p* = 0.396; Fig 1E).

**Fig 2:**
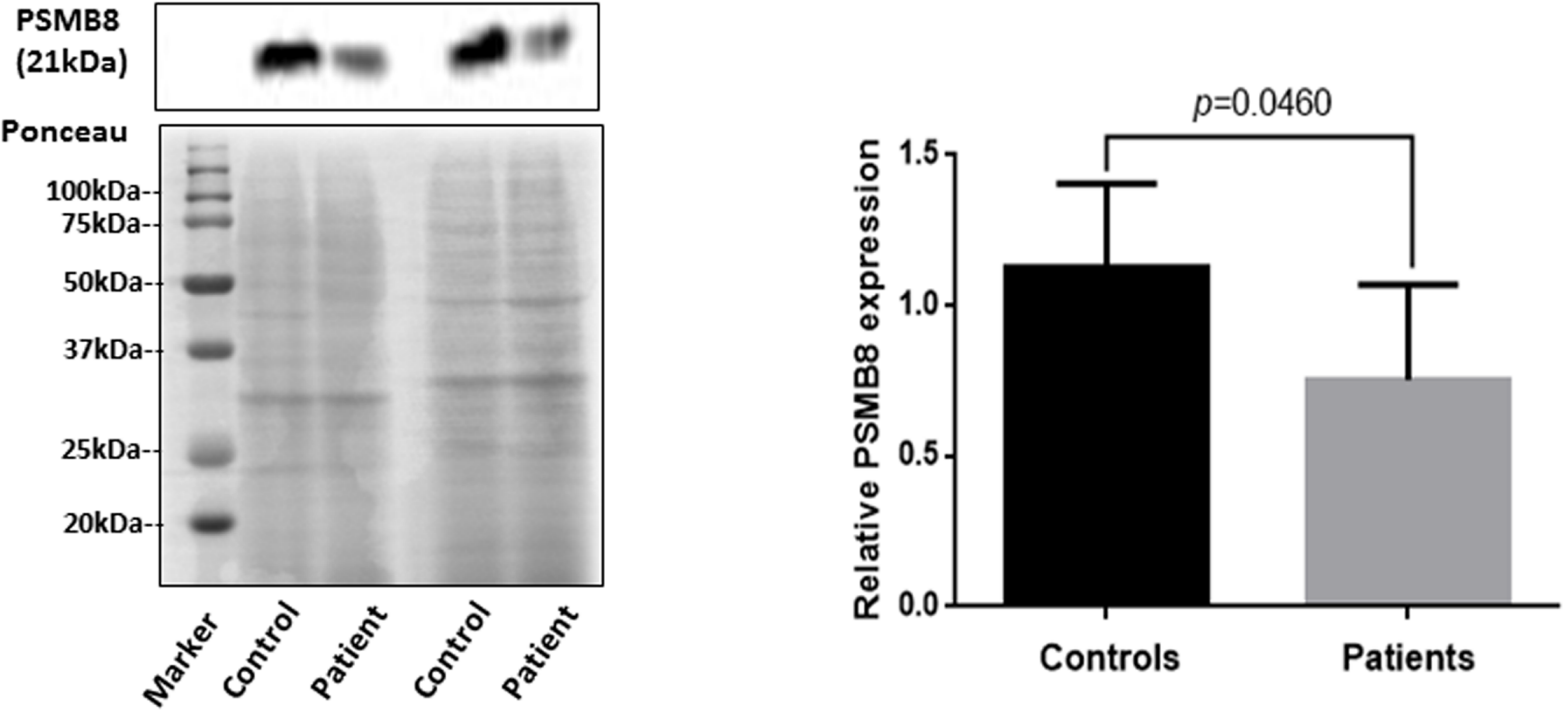
Analysis of PSMB8 protein expression. Western blot analysis in PBMCs of healthy controls (n = 6) and patients with active GV (n = 7) revealed significant decrease (*p* = 0.0460) in expression of PSMB8 after normalization with Ponceau staining.

Furthermore, the decreased transcript expression of *PSMB8* in patients with vitiligo was confirmed at protein level by western blot analysis in PBMCs of healthy controls (n = 6) and patients with active GV (n = 7). A significant decrease (*p* = 0.0460) in expression of PSMB8 was observed in patients as compared to controls (Fig 2).

### Genotype—phenotype correlation for *PSMB8* rs2071464 polymorphism

Further, the expression of *PSMB8* transcripts was analyzed with respect to *PSMB8* rs2071464 genotypes. Interestingly, *PSMB8* transcript levels were significantly reduced in individuals with the susceptible CC genotype when compared with CT and TT genotypes (*p* = 0.009 and *p* = 0.003, respectively; Fig 1F). However, no significant difference in *PSMB8* transcripts levels was observed between individuals with the CT and TT genotypes (Fig 1F).

### *TAP1* transcript levels in vitiligo

**Fig 3:**
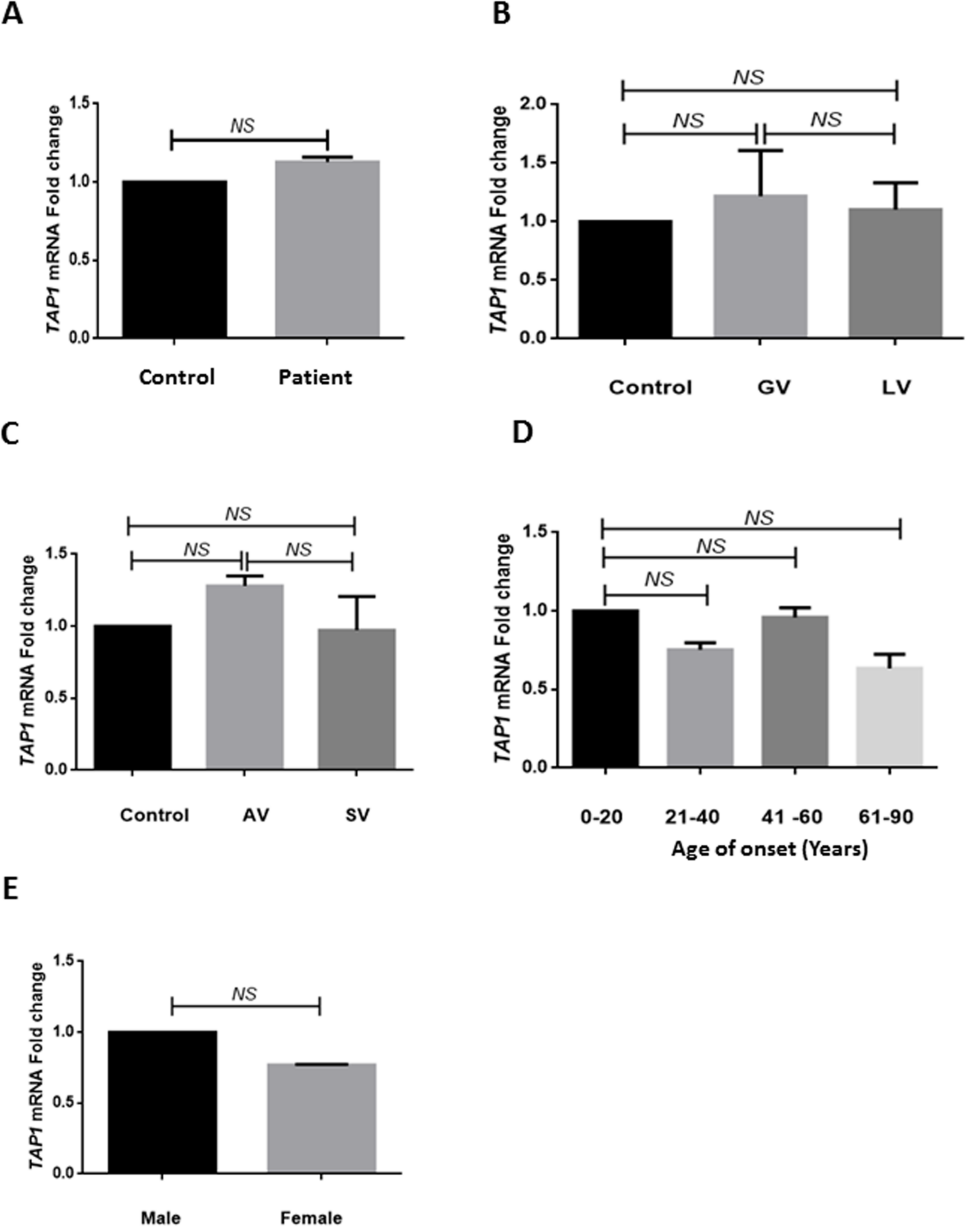
Relative gene expression of *TAP1* in patients and controls. (A) Expression of *TAP1* transcripts in 96 controls, 91 patients with vitiligo was analyzed by applying unpaired t-test. No significant difference in transcript levels of *TAP1* was observed as compared to controls (mean ΔCp ± SEM 5.59 ± 0.188 vs 5.421 ± 0.228; *p* = 0.553). Expression of *TAP1* transcripts in controls and patients with vitiligo showed approximately 1.12-fold change (NS) as determined by the 2^-ΔΔCp^ method. (B) Expression of *TAP1* transcripts in 96 controls and 72 patients with GV and 19 patients with LV was analyzed by using one-way ANOVA. Patients with GV and LV showed no significant difference in *TAP1* transcript levels as compared with controls (*p* = 0.856 and *p* = 0.090, respectively). No significant difference in *TAP1* transcript levels was observed between GV and LV (*p* = 0.219). (C) Expression of *TAP1* transcripts in 96 controls and 69 patients with AV and 22 patients with SV was analyzed by using one-way ANOVA. Patients with AV and SV showed no significant difference in *TAP1* transcripts levels as compared with controls (*p* = 0.671 and *p* = 0.291, respectively). No significant difference in *TAP1* transcript levels was observed among patients with AV and SV (*p* = 0.634). (D) Expression of *TAP1* transcripts with respect to different age of onset groups in 91 patients with vitiligo was analyzed by using one-way ANOVA. No significant difference in *TAP1* transcripts levels was observed in patients with respect to different age of onset groups. (F) Expression of *TAP1* transcripts with respect to sex differences in 48 male patients and 43 female patients was analyzed by applying unpaired t-test. No significant difference was observed in both the groups (*p* = 0.444).

Analysis of *TAP1* transcript levels was carried out after normalization with *GAPDH* expression. No significant difference in expression of *TAP1* transcripts was observed (*p* = 0.553) between patients and controls (Fig 3A). The 2^-ΔΔCp^ analysis showed approximately 1.12- fold change in expression of *TAP1* transcript in patients as compared to controls (Fig 3A). Analysis based on type of the disease suggested no significant difference in *TAP1* transcript levels in patients with GV and LV in comparison to controls (*p* = 0.090 and *p* = 0.219 respectively; Fig 3B). Moreover, there was no significant difference in patients with AV and SV as compared to controls (*p* = 0.671 and *p* = 0.291 respectively; Fig 3C). When expression of *TAP1* transcripts was monitored in different age at onset groups of patients, no significant difference was observed in any of the age of onset groups i.e., 21–40, 41–60 and 61–80 years when compared with 1–20 years (Fig 3D). Gender-based analysis showed no significant difference in *TAP1* transcripts in both the groups (Fig 3F).

### Bioinformatics analyses

Analysis of functional consequences of *PSMB8* rs2071464 by RegulomeDB was scored 6 and classified as having minimal binding evidence (Table 5). HaploReg v4.1 predicted *PSMB8* rs2071464 could alter 7 DNA motifs. RegulomeDB revealed that the Chromatin state is altered favoring strong transcription and genic enhancer by the polymorphism in peripheral blood cells (http://www.regulomedb.org/snp/chr6/32809075). Analysis by HaploReg v4.1 further confirmed the enhancer chromatin state in peripheral blood and T cells due to the polymorphism (http://archive.broadinstitute.org/mammals/haploreg/detail_v4.1.php?query=&id=rs2071464).

**Table 5:** *In silico* prediction results for *PSMB8* rs2071464 polymorphism.

SNP ID	Gene Symbol	SNP Location	Chromosomal Location	Regulome DB Score/ Prediction	HaploRedv4.1Motifs changed by SNP	Tissue
rs2071464	PSMB8	Intron 6	chr6:32809075	6 / Minimal binding Evidence	7 altered motifs	Peripheral Blood

*TAP1* exon 10 A>G leads to variation in TAP1 protein from Asp to Gly at position 637 [36]. PANTHER tool showed variation Asp to Gly at position 637 is not deleterious for TAP1 function, with the score of 0.3456 (Table 5). POLYPHEN tool showed that the substitution does not affect the phenotype or have damaging effects on the function of TAP1 protein. I-MUTANT and MUPRO predictions revealed decreased stability of Asp637Gly variants compared to native structure, which might affect the protein function. SNPs AND GO tool revealed that the variant doesn’t show disease like trait. (Table 6).

**Table 6:** *In silico* prediction results for *TAP1* rs1135216 polymorphism.

Amino acid change	SIFT	PANTHER	SNPs and GO	POLYPHEN	I-MUTANT	I-MUTANT Score	MUPRO
Asp637Gly	Tolerated	0.34565	Neutral	Benign	Decrease	-1.00	Decrease

**SIFT**: Sorting Intolerant From Tolerant; **PANTHER**: Protein Analysis Through Evolutionary Relationships; **SNPs and GO**: Single Nucleotide Polymorphisms and Gene Ontology; **PolyPhen**: Polymorphism Phenotyping.

## Discussion

The association of MHC region has been implicated in several GWAS on vitiligo including in Indian subcontinent [16,17,19,37–42]. Association of MHC class II region with generalized vitiligo was reported in European-derived white population by Jin *et al*., [42]. The strong link between autoimmune diseases and MHC class II genes suggests that abnormalities in MHC class II gene products may play a crucial role in vitiligo susceptibility. Interestingly, the association of GV with SNPs in the *PSMB8-TAP1* region of the MHC has been reported to derive from LD with primary association signals in the MHC class I and class II regions [17]. Any alterations in function or expression of *PSMB8* or *TAP1* proteins could potentially affect the antigenic repertoire expressed on the cell surface and may alter peripheral tolerance [43]. Several studies have addressed the association of *PSMB8* and *TAP1* polymorphisms in patients with vitiligo (Table 7); however, studies revealing the impact of these polymorphisms at transcript and protein levels are few.

**Table 7:** Genetic association studies on *PSMB8* and *TAP1* polymorphisms in Vitiligo.

Sr. No.	Gene	SNP	Population	Association	Reference
1.	*PSMB8*	rs2071543	Western	No	[14]
		rs2071543	Indian	Yes	[34]
		rs2071627	Western	No	[17]
		rs2071464	Western	Yes	[14]
			Egyptian	No	[44]
			Saudi	No	[22]
			Western	No	[14]
2.	*TAP1*	Intron7 C/T	Western	Yes	[14]
			Saudi	Yes	[22]
		rs1135216	Saudi	Yes	[45]

The present study suggests the association of *PSMB8* rs2071464 SNP with GV as well as with the disease activity (AV); however, *TAP1* rs1135216 SNP was not associated with vitiligo in Gujarat. Our results are in accordance with the previous study [14] reported in Western population for *PSMB8* SNP. In contrast, two studies have found *TAP1* exon 10 SNP to be associated with vitiligo in Saudi population, and this may be due to differences in the ethnicity [22,45]. Birlea *et al*., [18] have addressed 34 SNPs spanning *TAP1-PSMB8* region in GWAS and the meta-analysis study in GV patients; however, no association was observed for *TAP1* rs1135216 and *PSMB8* rs2071627 SNPs.

The *PSMB8* encodes IFN-γ inducible subunit (b5i/LMP7) of the immunoproteasome, which degrades the ubiquitin-tagged cytoplasmic proteins into peptides that are especially suited for presentation by MHC class I molecules to CD8^+^ cytotoxic T cells [46]. Significant association of *PSMB8* rs2071464 leads us to speculate some functional consequences of this SNP in the disease pathogenesis. Intriguingly, the decreased expression was associated with the susceptible ‘C’ allele of *PSMB8* rs2071464; however, the mechanism is not yet clear. *In silico* prediction tools have predicted that *PSMB8* rs2071464 C>T variation might alter chromatin to enhancer state and result in induced gene expression in peripheral blood cells. Recent studies have explored that several of *cis*-regulatory SNPs could affect histone modifications and change chromatin state transition from repressor to enhancer state [47]. Our results correlate with these findings as higher expression of PSMB8 was observed in individuals having variant ‘TT’ genotype as compared to ‘CC’ genotype (Fig 1). A significant decrease in transcript as well as protein expression of *PSMB8* in PBMCs of patients with GV and AV is revealed in the present study. Our findings have recently been supported by the blood transcriptomics analysis of vitiligo patients which revealed significant down regulation of *PSMB8* expression in patients [48]. In addition, another recent study has demonstrated the IFN-γ induced lower expression of *PSMB8* in PBMCs of vitiligo patients as compared to controls [34].

Moreover, it has been observed that the down-regulation of *PSMB8* expression leads to suppression of MHC class I molecule surface expression [49]. In addition, the IFN-γ induced immunoproteasomes have been associated with the improved processing of MHC class I antigens [50]. It has been reported that the presentation of a majority of MHC class I epitopes was strikingly reduced in immunoproteasome-deficient mice [51]. Moreover, Xu *et al*., [52] have also reported a significant decrease of 26S proteasome in lesions of vitiligo patients. Thus, the decreased expression of *PSMB8* in the present study, in conjunction with the above-discussed studies advocates the possibility of reduced MHC class I molecules in the patients and indicates the crucial role of *PSMB8* in vitiligo immunopathogenesis.

Autoimmune diseases are characterized by decreased expression of MHC class I on lymphocytes [53]. The appropriate MHC class I expression is necessary for self-tolerance, and abnormalities in such expression may lead to autoimmunity [54]. Zaiss *et al*., [55] have reported that proteasome immuno-subunits protect against the development of CD8^+^ T-cell mediated autoimmune diseases. They showed that mice deficient for the immune-subunits β5i/LMP7 and β2i/MECL-1 develop early-stage multi-organ autoimmunity following irradiation [55]. Several reports including ours have suggested a decreased CD4^+^⁄CD8^+^ ratio in vitiligo patients, indicating the prevalence of CD8^+^ cells in patients [56–58]. Thus, a decrease in immunoproteasome levels may lead to a breakdown of self-tolerance, resulting in an increase of CD8^+^ T cells directed towards melanocytes in predisposed individuals which could not be checked upon by the insufficient numbers and functionally deficient regulatory T cells (Tregs) in patients with vitiligo [58,59].

Transport of antigenic peptides across ER membrane is mediated by TAP1 and TAP2 molecules [60]. We did not find a significant association of *TAP1* rs1132516 SNP with vitiligo, as well as there was no difference in *TAP1* transcript levels between cases and controls. The ‘G’ allele occurred predominantly in AV patients compared to SV however, it was considered non-significant due to Bonferroni’s correction. The higher frequency of ‘G’ allele in AV patients indicates its involvement in the autoimmune basis of vitiligo. The bioinformatics analysis revealed that *TAP1* rs1135216 SNP (Asp637Gly) leads to a decrease in the stability of TAP1 protein. Moreover, it has been reported that the polymorphism in *TAP1* gene product did not show any measurable change in protein function but has an influence on peptide selectivity [36]. The binding of antigenic peptides to class I molecules depends on both length (usually 8–10 residues) and sequence [61]. The specificity of these reactions and their biological functions are affected by the 3D conformation of the peptide, HLA complexes, compatibility of the peptide sequence with its HLA class I binding pocket etc [62]. Interestingly, significant differences in the amino-acid signatures of the peptide-binding pockets of MHC class I α chains as well as class II β chains were observed between vitiligo patients and unaffected controls [15]. Though *TAP1* SNP was not associated with vitiligo but the predominant presence of ‘G’ allele in combination with other SNPs in this region might affect the peptide selectivity in patients. *PSMB8* polymorphism in addition to previously reported susceptibility loci such *TNFA*, *TNFB*, *IL1B*, *IFNG*, *NALP1*, *IL4* etc. demonstrate immunogenetic predisposition in vitiligo patients from Gujarat [7, 63–67]. Overall, studies implicate a break in immunological tolerance in vitiligo. A similar type of etiopathology has been observed in alopecia areata (a common autoimmune disorder that often results in unpredictable hair loss). The melanocyte is the main autoimmune target in both the disorders. Both are IFN-γ dependent and shares common immunogenetic loci such as *AIRE*, *CTLA4*, *NALP1*, and MHC region [68–72]. Surprisingly, the co-occurrence of vitiligo and alopecia areata is rare [73]. Unequal expression of MHC class I and II might be a base for the reverse correlation between the incidents of vitiligo and alopecia areata [73]. Hence, the genes involved in antigen processing might have a role in the breakdown of immune tolerance and precipitation of vitiligo.

## Conclusion

In conclusion, the association of *PSMB8* rs2071464 polymorphism with generalized and active vitiligo suggests defective antigen processing which might influence the peptide repertoire presented to the immune cells targeting melanocytes. However, further replicative studies and *in vitro* functional studies for *PSMB8* and *TAP1* are needed to delineate the role of defective antigen processing and presentation pathways in vitiligo pathogenesis.

## Supporting information

S1 Text Bioinformatics analysis. (DOC)

S1 Table Demographic characteristics of patients with vitiligo and controls. (DOCX)

S2 Table Primers used for genotyping of *PSMB8* rs2071464 and *TAP1* rs1135216 SNPs. (DOCX)

S3 Table Primers used for Sequencing of *PSMB8* and *TAP1* SNP*s*. (DOCX)

S4 Table Primers used for gene expression of *PSMB8* and *TAP1*. (DOCX)

S1 Fig (A) PCR-RFLP analysis of *PSMB8* rs2071464 SNP on 3.5% agarose gel: Lane M shows 50bp DNA ladder, lanes: 1 & 2 show homozygous (CC) genotypes; lanes: 3 & 6 show homozygous (TT) genotypes and lanes: 3 & 6 show heterozygous (CT) genotypes. (B) ARMS-PCR analysis of *TAP1* rs1135216 SNP on 3.5% agarose gel: Lane M shows 50bp DNA ladder, lanes: 1, 2 & 3, 4 show homozygous (AA) genotypes; lanes: 5, 6 shows heterozygous (AG) genotype and lanes: 7, 8 shows homozygous (GG) genotype.
(TIF)

S2 Fig Confirmation of genotyping results of *PSMB8* rs2071464 SNP by sequencing of PCR products. **A)** *PSMB8* rs2071464 CC genotype, **B)** *PSMB8* rs2071464 CT genotype, **C)** *PSMB8* rs2071464 TT genotype.
(TIF)

S3 Fig Confirmation of genotyping results of *TAP1* rs1135216 SNP by sequencing of PCR products. **A)** *TAP1* rs1135216 AA genotype, **B)** TAP1 rs1135216 AG genotype, **C)** TAP1 rs1135216 GG genotype.
(TIF)

## References

[1] Kruger and Schallreuter. A review of the worldwide prevalence of vitiligo in children adolescents and adults. Int J Dermatol. 2012; 51: 1206–1212. doi: 10.1111/j.1365-4632.2011.05377.x 22458952 10.1111/j.1365-4632.2011.05377.x

[2] Handa S and Kaur I. Vitiligo: clinical findings in 1436 patients. J Dermatol. 1999; 10: 653–657. 10.1111/j.1346-8138.1999.tb02067.x 10554431

[3] Valia AK, Dutta PK. Textbook and Atlas of Dermatology (Indian Association of Dermatologists, Venereologists and Leprologists), Bombay: Bhalani Publishing House; 1996 pp. 500–586.

[4] Shajil EM, Chatterjee S, Agrawal D, Bagchi T, Begum R. Vitiligo: pathomechanisms and genetic polymorphism of susceptible genes. Indian J Exp Biol. 2006; 44:526–39. 16872041

[5] Alkhateeb A, Fain PR, Thody A, Bennett DC, Spritz RA. Epidemiology of vitiligo and associated autoimmune diseases in Caucasian probands and their families. Pigment Cell Res. 2003; 16: 208–214. 12753387 10.1034/j.1600-0749.2003.00032.x

[6] Laddha NC, Dwivedi M, Mansuri MS, Singh M, Gani AR, Yeola AP, et al., Role of oxidative stress and autoimmunity in onset and progression of vitiligo. Exp Dermatol. 2014; 23: 352–353. doi: 10.1111/exd.12372 24628992 10.1111/exd.12372

[7] Laddha NC, Dwivedi M, Mansuri MS, Singh M, Patel HH, Agarwal N, et al. Association of neuropeptide Y (NPY), interleukin-1B (IL1B) genetic variants and correlation of IL1B transcript levels with vitiligo susceptibility. PLoS One. 2014; 9:e107020 doi: 10.1371/journal.pone.0107020 25221996 10.1371/journal.pone.0107020 PMC4164539

[8] van den Boorn JG, Konijnenberg D, Dellemijn TA, van der Veen JP, Bos JD, Melief CJ et al Autoimmune destruction of skin melanocytes by perilesional T cells from vitiligo patients. J Invest Dermatol. 2009; 129: 2220–2232. doi: 10.1038/jid.2009.32 19242513 10.1038/jid.2009.32

[9] Dwivedi M, Laddha NC, Arora P, Marfatia YS, Begum R. Decreased regulatory T-cells and CD4 (+)/ CD8(+) ratio correlate with disease onset and progression in patients with generalized vitiligo. Pigment Cell Melanoma Res. 2013; 26: 586–591. doi: 10.1111/pcmr.12105 23574980 10.1111/pcmr.12105

[10] Uebel S, Tampé R. Specificity of the proteasome and the TAP transporter. Curr Opin Immunol. 1999; 11: 203–208. 10322157 10.1016/s0952-7915(99)80034-x

[11] Saiah ID, Zucman SC, Schmitz J, Chaves-Vieira ML, Bach JF. Polymorphism of antigen processing (TAP, LMP) and HLA class II genes in celiac disease. Hum Immunol. 1994; 40: 8–16. 8045794 10.1016/0198-8859(94)90015-9

[12] Kumagai S, Kanagawa S, Morinobu A, Takada M, Nakamura K, Sugai S, et al. Association of a new allele of the TAP2 gene, TAP2*Bky2 (Val577), with susceptibility to Sjögren's syndrome. Arthritis Rheum. 1997; 40: 1685–1692. doi: 10.1002/1529-0131(199709)40:9&lt;1685::AID-ART19&gt;3.0.CO;2-I 9324024 10.1002/art.1780400919

[13] Teisserenc H, Schmitt W, Blake N, Dunbar R, Gadola S, Gross WL, et al. A case of primary immunodeficiency due to a defect of the major histocompatibility gene complex class I processing and presentation pathway. Immunol Lett. 1997; 57: 183–187. 9232449 10.1016/s0165-2478(97)00072-2

[14] Casp CB, She JX, McCormack WT. Genes of the LMP/TAP cluster are associated with the human autoimmune disease vitiligo. Genes Immun. 2003; 4: 492–499. doi: 10.1038/sj.gene.6364016 14551602 10.1038/sj.gene.6364016

[15] Krämer U, Illig T, Grune T, Krutmann J, Esser C. Strong associations of psoriasis with antigen processing LMP and transport genes TAP differ by gender and phenotype. Genes Immun. 2007; 8: 513–517. doi: 10.1038/sj.gene.6364404 17581627 10.1038/sj.gene.6364404

[16] Singh A, Sharma P, Kar HK, Sharma VK, Tembhre MK, Gupta S, et al. HLA alleles and amino acid signatures of the peptide binding pockets of HLA molecules in Vitiligo. J. Invest. Dermatol. 2012; 132: 124–134. doi: 10.1038/jid.2011.240 21833019 10.1038/jid.2011.240

[17] Birlea SA, Ahmad FJ, Uddin RM, Ahmad S, Pal SS, Begum R et al. Association of generalized vitiligo with MHC class II loci in patients from the Indian subcontinent. J Invest Dermatol. 2013; 133: 1369–1372. doi: 10.1038/jid.2012.501 23303446 10.1038/jid.2012.501 PMC3626744

[18] Birlea SA, Jin Y, Bennett DC, Herbstman DM, Wallace MR, McCormack WT et al. Comprehensive association analysis of candidate genes for generalized vitiligo supports *XBP1*, *FOXP3*, and *TSLP*. J Invest Dermatol. 2011; 131: 371–381. doi: 10.1038/jid.2010.337 21085187 10.1038/jid.2010.337 PMC3172683

[19] Jin Y, Andersen G, Yorgov D, Ferrara TM, Ben S, Brownson KM, et al, Genome-wide association studies of autoimmune vitiligo identify 23 new risk loci and highlight key pathways and regulatory variants. Nat Genet. 2016; 48:1418–1424. doi: 10.1038/ng.3680 27723757 10.1038/ng.3680 PMC5120758

[20] Song R, Harding CV. Roles of proteasomes, transporter for antigen presentation (*TAP*), and beta 2-microglobulin in the processing of bacterial or particulate antigens via an alternate class I MHC processing pathway. J Immunol. 1996; 156: 4182–4190. 8666786

[21] Koopmann JO, Hämmerling GJ, Momburg F. Generation, intracellular-transport and loading of peptides associated with MHC class-l molecules. Curr Opin immune. 1997; 9: 80–88. 10.1016/s0952-7915(97)80163-x 9039771

[22] Babalghith A. *TAP1* and *LMP7* Gene Polymorphisms Associated with Vitiligo in Saudi Community. Int J Curr Microbiol App Sci. 2014; 3: 1–9.

[23] Ezzedine K, Lim HW, Suzuki T, Katayama I, Hamzavi I, Lan CC, et al, Revised classification/nomenclature of vitiligo and related issues: the Vitiligo Global Issues Consensus Conference. Pigment Cell Melanoma Res. 2012; 25: E1–13. 10.1111/j.1755-148X.2012.00997.x PMC3511780 22417114

[24] Falabella R, Arrunategui A, Barona MI, et al, The minigrafting test for vitiligo: detection of stable lesions for melanocyte transplantation. J Am Acad Dermatol. 1995; 32: 228–232. 7829707 10.1016/0190-9622(95)90131-0

[25] Shi YY, He L, SHEsis, a powerful software platform for analyses of linkage disequilibrium, haplotype construction, and genetic association at polymorphism loci, Cell Res. 2005; 15: 97–98. doi: 10.1038/sj.cr.7290272 15740637 10.1038/sj.cr.7290272

[26] Ward LD, Kellis M. HaploReg: a resource for exploring chromatin states, conservation, and regulatory motif alterations within sets of genetically linked variants. Nucleic acids research. 2011; 40: 930–934. 10.1093/nar/gkr917 PMC3245002 22064851

[27] Boyle AP, Hong EL, Hariharan M, Cheng Y, Schaub MA, Kasowski M, Karczewski KJ, Park J, Hitz BC, Weng S, Cherry JM. Annotation of functional variation in personal genomes using RegulomeDB. Genome research. 2012; 22: 1790–1797. doi: 10.1101/gr.137323.112 22955989 10.1101/gr.137323.112 PMC3431494

[28] Kumar P, Henikoff S, Ng PC, Predicting the effects of coding non-synonymous variants on protein function using the SIFT algorithm, Nat. Protoc. 2009; 4: 1073–1081. doi: 10.1038/nprot.2009.86 19561590 10.1038/nprot.2009.86

[29] Thomas PD, Campbell MJ, Kejariwal A, Mi H, Karlak B, Daverman R, et al, PANTHER: a library of protein families and subfamilies indexed by function, Genome Res. 2003; 13: 2129–2141. doi: 10.1101/gr.772403 12952881 10.1101/gr.772403 PMC403709

[30] Capriotti E, Fariselli P, Rossi I, Casadio R. A three-state prediction of single point mutations on protein stability changes. BMC Bioinformatics. 2008; 9: S2–S6. 10.1186/1471-2105-9-S2-S6 PMC2323669 18387208

[31] Adzhubei A, Schmidt S, Peshkin L, Ramensky VE, Gerasimova A, Bork P, et al, A method and server for predicting damaging missense mutations, Nat Methods. 2010; 7: 248–249. doi: 10.1038/nmeth0410-248 20354512 10.1038/nmeth0410-248 PMC2855889

[32] Cheng J, Randall A, Baldi P. Prediction of protein stability changes for single-site mutations using support vector machines, Proteins. 2006; 62: 1125–1132. doi: 10.1002/prot.20810 16372356 10.1002/prot.20810

[33] Capriotti E, Calabrese R, Casadio R, Predicting the insurgence of human genetic diseases associated to single point protein mutations with support vector machines and evolutionary information, Bioinformatics. 2006; 22: 2729–2734. doi: 10.1093/bioinformatics/btl423 16895930 10.1093/bioinformatics/btl423

[34] Dani P, Patnaik N, Singh A, Jaiswal A, Agrawal B, Kumar AA, et al, Association and expression of antigen processing gene PSMB8 coding for Low Molecular Mass Protease 7 (LMP7) with Vitiligo in North India: case‐control study. British Journal of Dermatology. 2017; doi: 10.1111/bjd.15391 28207947 10.1111/bjd.15391

[35] Deng GY, Muir A, Maclaren NK, She JX. Association of LMP2 and LMP7 genes within the major histocompatibility complex with insulin-dependent diabetes mellitus: Population family studies. Am J Hum Genet. 1995; 56: 528–534. 7847389 PMC1801142

[36] Quadri SA, Singal DP. Peptide transport in human lymphoblastoid and tumor cells: effect of transporter associated with antigen presentation (TAP) polymorphism. Immunol Lett. 1998; 61: 25–31. 9562372 10.1016/s0165-2478(97)00157-0

[37] Fernando MM, Stevens CR, Walsh EC, De Jager PL, Goyette P, Plenge RM, et al Defining the role of the MHC in autoimmunity: a review and pooled analysis. PLoS Genet. 2008; 4 e1000024 doi: 10.1371/journal.pgen.1000024 18437207 10.1371/journal.pgen.1000024 PMC2291482

[38] deVijlder HC, Westerhof W, Schreuder GM, de Lange P, Claas FH. Difference in pathogenesis between vitiligo vulgaris and halo nevi associated with vitiligo is supported by an HLA association study. Pigment Cell Res. 2004; 17: 270–274. doi: 10.1111/j.1600-0749.2004.00145.x 15140072 10.1111/j.1600-0749.2004.00145.x

[39] Zhang XJ, Liu HS, Liang YH, Sun LD, Wang JY, Yang S, et al Association of HLA class I alleles with vitiligo in Chinese Hans. J Dermatol Sci. 2004; 35: 165–168. doi: 10.1016/j.jdermsci.2004.05.003 15265531 10.1016/j.jdermsci.2004.05.003

[40] Quan C, Ren YQ, Xiang LH, Sun LD, Xu AE, Gao XH, et al Genome-wide association study for vitiligo identifies susceptibility loci at 6q27 and the MHC. Nat Genet. 2010; 42: 614–618. doi: 10.1038/ng.603 20526339 10.1038/ng.603

[41] Jin Y, Birlea SA, Fain PR, Gowan K, Riccardi SL, Holland PJ, et al Variant of TYR and autoimmunity susceptibility loci in generalized vitiligo. New Eng J Med. 2010; 362: 1686–1697. doi: 10.1056/NEJMoa0908547 20410501 10.1056/NEJMoa0908547 PMC2891985

[42] Jin Y, Birlea SA, Fain PR, Gowan K, Riccardi SL, Holland PJ et al Genome-Wide Analysis Identifies a Quantitative Trait Locus in the MHC Class II Region Associated with Generalized Vitiligo Age of Onset. J Invest Dermatol. 2011; 131: 1308–1312. doi: 10.1038/jid.2011.12 21326295 10.1038/jid.2011.12 PMC3172680

[43] Groettrup M, Khan S, Schwarz K, Schmidtke G. Interferon-gamma inducible exchanges of 20S proteasome active site subunits: why? Biochimie. 2001; 83: 367–372. 11295499 10.1016/s0300-9084(01)01251-2

[44] Seif Eldin NS, Teama S, Amro K, Farag HM, Nour Eldin SM, Elhawary NA. Polymorphisms of TAP1/LMP7 loci in Egyptian patients with vitiligo. Egypt J Med Hum Genet. 2006;7:241–249.

[45] Elhawary NA, Bogari N, Jiffri EH, Rashad M, Fatani A, Tayeb M. Transporter TAP1-637G and Immunoproteasome PSMB9-60H Variants Influence the Risk of Developing Vitiligo in the Saudi Population. Disease Markers. 2014: 2014; 260732 doi: 10.1155/2014/260732 25548428 10.1155/2014/260732 PMC4273470

[46] Basler M, Kirk CJ, Groettrup M. The immunoproteasome in antigen processing and other immunological functions. Curr Opin Immunol. 2013; 25: 74–80. doi: 10.1016/j.coi.2012.11.004 23219269 10.1016/j.coi.2012.11.004

[47] Taudt A, Colomé-Tatché M, Johannes F. Genetic sources of population epigenomic variation. Nature Reviews Genetics. 2016; doi: 10.1038/nrg.2016.45 27156976 10.1038/nrg.2016.45

[48] Dey-Rao R, Sinha AA. Vitiligo blood transcriptomics provides new insights into disease mechanisms and identifies potential novel therapeutic targets. BMC genomics. 2017; 18:109 doi: 10.1186/s12864-017-3510-3 28129744 10.1186/s12864-017-3510-3 PMC5273810

[49] Seliger B, Maeurer MJ and Ferrone S. Antigen-processing machinery breakdown and tumor growth. Immunol Today. 2000; 9: 455–464. 10.1016/s0167-5699(00)01692-3 10953098

[50] Seifert U, Bialy LP, Ebstein F, Bech-Otschir D, Voigt A, Schröter F, et al Immunoproteasomes preserve protein homeostasis upon interferon-induced oxidative stress. Cell. 2010; 142: 613–624. doi: 10.1016/j.cell.2010.07.036 20723761 10.1016/j.cell.2010.07.036

[51] Kincaid EZ, Che JW, York I, Escobar H, Reyes-Vargas E, Delgado JC, et al Mice completely lacking immunoproteasomes show major changes in antigen presentation. Nat Immunol. 2011; 13: 129–135. doi: 10.1038/ni.2203 22197977 10.1038/ni.2203 PMC3262888

[52] Xu W, Lin FQ, Liu JF, Fu LF, Hong WS, Zhou MN, et al Impact on tyrosinase expression and export from endoplasmic reticulum by inhibition of 26S proteasome. Zhonghua Yi Xue Za Zhi. 2013; 93: 123–127. 23648349

[53] Fu Y, Yan G, Shi L, Faustman D. Antigen processing and autoimmunity. Evaluation of mRNA abundance and function of HLA-linked genes. Ann NY Acad Sci. 1998; 42: 138–155. 10.1111/j.1749-6632.1998.tb09642.x 9599304

[54] Fu Y, Nathan DM, Li F. Defective major histocompatibility complex class I expression on lymphoid cells in autoimmunity. J Clin Invest. 1993; 91: 2301–2307. doi: 10.1172/JCI116459 8486790 10.1172/JCI116459 PMC288235

[55] Zaiss DMW, Bekker CP, Gröne A, Lie BA, Sijts AJ. Proteasome immunosubunits protect against the development of CD8 T-cell-mediated autoimmune diseases. J Immunol. 2011; 187: 2302–2309. doi: 10.4049/jimmunol.1101003 21804012 10.4049/jimmunol.1101003 PMC3159700

[56] Grimes PE, Ghoneum M, Stockton T, Payne C, Kelly AP, Alfred L. T cell profiles in vitiligo. J Am Acad Dermatol. 1986; 14: 196–201 2936773 10.1016/s0190-9622(86)70021-2

[57] Halder RM, Walters CS, Johnson BA, Chakrabarti SG, Kenney JA Jr. Aberrations in T lymphocytes and natural killer cells in vitiligo: a flow cytometric study. J Am Acad Dermatol. 1986; 14: 733–737 2940268 10.1016/s0190-9622(86)70085-6

[58] Dwivedi M, Laddha NC, Arora P, Marfatia YS, Begum R. Decreased regulatory T-Cells and CD4+/CD8+ ratio correlate with disease onset and progression in patients with generalized vitiligo. Pigment Cell Melanoma Res. 2013; 26: 586–591. doi: 10.1111/pcmr.12105 23574980 10.1111/pcmr.12105

[59] Dwivedi M, Kemp EH, Laddha NC, Mansuri MS, Weetman AP, Begum R. Regulatory T cells in Vitiligo: Implications for pathogenesis and therapeutics. Autoimmun Rev. 2015; 14: 49–56. doi: 10.1016/j.autrev.2014.10.002 25308528 10.1016/j.autrev.2014.10.002

[60] Monoco JJ. A molecular model of MHC class-I-restricted antigen processing. Immunol Today. 1992; 13: 173–179. doi: 10.1016/0167-5699(92)90122-N 1386516 10.1016/0167-5699(92)90122-N

[61] vanBleek GM, Nathenson SG. Isolation of an endogenously processed immunodominant viral peptide from the class I H-2Kb molecule. Nature. 1990; 348: 213–216. doi: 10.1038/348213a0 1700303 10.1038/348213a0

[62] Engelhard VH. Structure of peptides associated with class I and class II MHC molecules. Annu Rev Immunol. 1994; 12: 181–207. doi: 10.1146/annurev.iy.12.040194.001145 7516668 10.1146/annurev.iy.12.040194.001145

[63] Laddha NC, Dwivedi M and Begum R (2012). Increased Tumor Necrosis Factor (TNF)-α and its promoter polymorphisms correlate with disease progression and higher susceptibility towards vitiligo. PLoS ONE. 7: e52298 doi: 10.1371/journal.pone.0052298 23284977 10.1371/journal.pone.0052298 PMC3527546

[64] Laddha NC, Dwivedi M, Gani AR, Mansuri MS, Begum R (2013). Tumor Necrosis Factor B (TNFB) genetic variants and its increased expression are associated with vitiligo susceptibility. PLoS ONE. 8: e81736 (IF 3.73) doi: 10.1371/journal.pone.0081736 24312346 10.1371/journal.pone.0081736 PMC3842287

[65] Dwivedi M, Laddha NC, Shah K, Shah BJ and Begum R (2013). Involvement of Interferon-Gamma (IFNG) Genetic Variants and Intercellular Adhesion Molecule-1 (ICAM1) in Disease Onset and Progression of Generalized Vitiligo. J. Interferon Cytokine Res. 33: 646–659. (IF 3.063) doi: 10.1089/jir.2012.0171 23777204 10.1089/jir.2012.0171 PMC3814581

[66] Dwivedi M, Laddha NC, Mansuri MS, Marfatia YS and Begum R (2013). Association of NLRP1 genetic variants and mRNA overexpression with generalized vitiligo and disease activity in a Gujarat population. Brit. J. Dermatol. 169:1114–1125. (IF 3.759) 23773036 10.1111/bjd.12467

[67] Imran M, Laddha NC, Dwivedi M, Mansuri MS, Singh J, Rani R, Gokhale RS, Sharma VK, Marfatia YS and Begum R (2012). Interleukin-4 genetic variants correlate with its transcript and protein levels in vitiligo patients. Brit. J. Dermatol. 167: 314–323 22512783 10.1111/j.1365-2133.2012.11000.x

[68] Harris JE. Viewpoint–Vitiligo and alopecia areata: Apples and oranges? Exp Dermatol. 2013; 22: doi: 10.1111/exd.12264 24131336 10.1111/exd.12264 PMC3867815

[69] Dwivedi M, Laddha NC, Imran M, Shah BJ and Begum R. Cytotoxic T-lymphocyte associated antigen-4 (CTLA-4) in isolated vitiligo: a genotype-phenotype correlation. Pigment Cell Melanoma Res. 2011; 24:737–740. doi: 10.1111/j.1755-148X.2011.00892.x 21794098 10.1111/j.1755-148X.2011.00892.x

[70] Petukhova L, Duvic M, Hordinsky M, Norris D, Price V, Shimomura Y, et al, Genome-wide association study in alopecia areata implicates both innate and adaptive immunity. Nature. 2010; 466: 113–117. doi: 10.1038/nature09114 20596022 10.1038/nature09114 PMC2921172

[71] Tazi-Ahnini R, McDonagh AJ, Wengraf DA, Lovewell TR, Vasilopoulos Y, Messenger AG, et al, The autoimmune regulator gene (AIRE) is strongly associated with vitiligo. Br J Dermatol. 2008; 159:591–596. doi: 10.1111/j.1365-2133.2008.08718.x 18616774 10.1111/j.1365-2133.2008.08718.x

[72] Gilhar A, Paus R and Kalish RS. Lymphocytes, neuropeptides, and genes involved in alopecia areata. J Clin Invest. 2007; 117:2019–2027. doi: 10.1172/JCI31942 17671634 10.1172/JCI31942 PMC1934574

[73] Dinh HV, Meyer KC, McCluskey J, Sinclair RD, Paus R. Differences in MHC expression between melanocytes of the hair follicle and epidermis. JID. 2007; 127: 2689 [Abstract].

