# Supplementary material for: A case-control study on association of proteasome subunit beta 8 (PSMB8) and transporter associated with antigen processing 1 (TAP1) polymorphisms and their transcript levels in vitiligo from Gujarat
Source: PLoS One. 2017 Jul 10;12(7):e0180958. doi: 10.1371/journal.pone.0180958 (PMC5507292; doi:10.1371/journal.pone.0180958)
Supplement: S1 Text — (DOC) [file pone.0180958.s001.doc]

**SUPPORTING INFORMATION**

**Bioinformatics analysis:**

***Sorting Intolerant From Tolerant (SIFT)***

SIFT predicts whether an amino acid substitution affects protein function. SIFT prediction is based on the degree of conservation of amino acid residues in sequence alignments derived from closely related sequences, collected through PSI-BLAST [1].

***I-MUTATNT***

I-Mutant 2.0 is a Support Vector Machine -based web server for the prediction of protein stability changes upon single-site mutations. I-Mutant uses the data set derived from ProTherm (Bava et al., 2004) that is presently the most comprehensive database of experimental data on protein mutations. I-Mutant 2.0 predicts whether the protein mutation stabilizes or destabilizes the protein in 77% of the cases when the protein sequence is available [2].

***Single Nucleotide Polymorphisms and Gene Ontology (SNPs & GO)***

SNPs & GO is a web server for the prediction of human disease-related single point protein mutations from the protein sequence. SNPs & GO is based on support vector machines, that uses different pieces of information, including that derived from the Gene Ontology annotation to predict if a given mutation can be classified disease-related or not, scoring with accuracy=82% and Matthews correlation coefficient=0.63 [3].

***Protein Analysis THrough Evolutionary Relationships (PANTHER)***

PANTHER estimates the likelihood that a specific amino acid substitution in a protein will affect the protein's function, by calculating the subPSEC (substitution position-specific evolutionary conservation) score based on an alignment of evolutionarily related proteins [4] and statistics from hidden Markov models [5,6].

***Polymorphism Phenotyping v2 (PolyPhen-2)***

**PolyPhen-2** is an automatic tool for prediction of possible impact of an amino acid substitution on the structure and function of a human protein. This prediction is based on a number of features comprising the sequence, phylogenetic and structural information characterizing the substitution [7]. PolyPhen-2 predicts the functional significance of an allele replacement from sequence and structure-based features of the substitution site, by Naïve Bayes classifier using supervised machine-learning.

***MUPRO***

MUPRO uses support vector machines to predict protein stability changes for single amino acid mutations leveraging both sequence and structural information. The sign of the stability changes is considered, only when the predictive method achieves 84% accuracy. The prediction accuracy obtained using sequence alone is close to the accuracy obtained using tertiary structure information [8].

**REFERENCES**

1. Kumar P, Henikoff S, Ng PC. Predicting the effects of coding non-synonymous variants on protein function using the SIFT algorithm. Nat Protoc. 2009; 4:1073-1081.
2. Capriotti E, Fariselli P Rossi I, Casadio R. A three-state prediction of single point mutations on protein stability changes. BMC Bioinformatics. 2008; 9:S2-S6.
3. Capriotti E, Calabrese R, Casadio R. Predicting the insurgence of human genetic diseases associated to single point protein mutations with support vector machines and evolutionary information. Bioinformatics. 2006; 22:2729–2734.
4. Thomas PD and Kejariwal A. Coding single-nucleotide polymorphisms associated with complex vs. Mendelian disease: Evolutionary evidence for differences e. PNAS. 2004; 101:15398-15403.
5. Thomas PD, Campbell MJ, Kejariwal A, Mi H, Karlak B, Daverman et al. PANTHER: a library of protein families and subfamilies indexed by function. Genome Res. 2003; 13:2129-2141.
6. Mi H, Poudel S, Muruganujan A, Casagrande JT, Thomas PD. PANTHER version 10: expanded protein families and functions, and analysis tools. Nucleic Acids Res. 2016; 44:D336-42.
7. Adzhubei IA, Schmidt S, Peshkin L, Ramensky VE, Gerasimova A, Bork P, et al. A method and server for predicting damaging missense mutations. Nat Methods. 2010; 7:248–249.
8. Cheng J, Randall A, Baldi P. Prediction of protein stability changes for single-site mutations using support vector machines. Proteins. 2006; 62:1125–1132.
