# Supplementary material for: A case-control study on association of proteasome subunit beta 8 (PSMB8) and transporter associated with antigen processing 1 (TAP1) polymorphisms and their transcript levels in vitiligo from Gujarat
Source: PLoS One. 2017 Jul 10;12(7):e0180958. doi: 10.1371/journal.pone.0180958 (PMC5507292; doi:10.1371/journal.pone.0180958)
Supplement: S1 Table — (DOCX) [file pone.0180958.s002.docx]

**Table S1.** Demographic characteristics of patients with vitiligo and controls.

|  | **Patients** |  | **Controls** |
| --- | --- | --- | --- |
| Average age  (mean age ± SD)  Sex: male  female  Onset age  (mean age ± SD)  Duration of disease  (mean ± SD)  Family history  **Type of disease**  Generalized  Localized  Active  Stable | (n =378) |  | (n =509) |
|  | 36.79 ± 18.66 yr  187 (49.47 %)  191 (50.53 %)  21.25 ± 12.53 yr  7.8 ± 6.9 yr  141 (37.30 %)  292  86  305  73 | 30.23 ± 14.46 yr  268 (52.68%)  241 (47.32%)  NA  NA  NA  NA  NA  NA  NA | |
|  |  |  |  |
