# Supplementary material for: A case-control study on association of proteasome subunit beta 8 (PSMB8) and transporter associated with antigen processing 1 (TAP1) polymorphisms and their transcript levels in vitiligo from Gujarat
Source: PLoS One. 2017 Jul 10;12(7):e0180958. doi: 10.1371/journal.pone.0180958 (PMC5507292; doi:10.1371/journal.pone.0180958)
Supplement: S2 Table — (DOCX) [file pone.0180958.s003.docx]

**Table S2:** Primers used for genotyping of *PSMB8* intron 6 rs2071464 and *TAP1* exon 10 rs1135216 SNPs.

| **SNP** | **Method** | **Primers** | **Product** | **RE** | **Cut products** |
| --- | --- | --- | --- | --- | --- |
| *PSMB8*  rs2071464 | PCR- RFLP | FP:5’-GCTATTCTGGAGGCGTTGTC-3’  RP:5’-AAGGAGTCTCACTCTGTGGC-3’ | 298bp | *Hha*I | 257bp, 41bp |
| *TAP1*  rs1135216 | ARMS-PCR | HGH FP:  5’-CTATGCTCCGCGCCCATCGT-3’  HGH RP:  5’-TGGGGAGAAGGCATCCACTCACG-3’  TAP1 FP-A:  5’- TTGCTCTGCAGAGGTAGA-3’  TAP1 FP-G:  5’- TTGCTCTGCAGAGGTAGG-3’  TAP1 RP:  5’-CACCTGTAACTGGCTGTTTG-3’ | HGH:  407bp  TAP1:  151bp | *­_* | _ |
