# Supplementary material for: A case-control study on association of proteasome subunit beta 8 (PSMB8) and transporter associated with antigen processing 1 (TAP1) polymorphisms and their transcript levels in vitiligo from Gujarat
Source: PLoS One. 2017 Jul 10;12(7):e0180958. doi: 10.1371/journal.pone.0180958 (PMC5507292; doi:10.1371/journal.pone.0180958)
Supplement: S3 Table — (DOCX) [file pone.0180958.s004.docx]

**Table S3:** Primers used for Sequencing of *PSMB8* and *TAP1* SNP*s.*

| **Gene** | **Primers** | **Product** |
| --- | --- | --- |
| *PSMB8* | FP: 5’-ACATGGGACTCGGCTCTCAGG-3’  RP: 5’- GAAGGCAGCAACAAGACACATGC -3’ | 591 bp |
| *TAP1* | FP: 5’- CTCATCTCACCACCCAGCCATC -3’  RP: 5’- TGCAGTGAGCCAAGATTGTGC -3’ | 746 bp |
