# Supplementary material for: A case-control study on association of proteasome subunit beta 8 (PSMB8) and transporter associated with antigen processing 1 (TAP1) polymorphisms and their transcript levels in vitiligo from Gujarat
Source: PLoS One. 2017 Jul 10;12(7):e0180958. doi: 10.1371/journal.pone.0180958 (PMC5507292; doi:10.1371/journal.pone.0180958)
Supplement: S4 Table — (DOCX) [file pone.0180958.s005.docx]

**Table S4:** Primers used for gene expression of *PSMB8* and *TAP1.*

| **Gene** | **Primers** | **Product** |
| --- | --- | --- |
| *GAPDH* | FP: 5’-ATCCCATCACCATCTTCCAGGA-3’  RP: 5’-CAAATGAGCCCCAGCCTTCT-3’ | 122 bp |
| *PSMB8* | FP: 5’ TCCTACATTAGTGCCTTACGGG 3’  RP: 5’- CAGATAGTACAGCCTGCATTCC -3’ | 135 bp |
| *TAP1* | FP: 5’- GGACCACTAGTATTTCAGGTATGC -3’  RP: 5’- GAGCAGTACCTCCACAGCC -3’ | 149 bp |
