# Supplementary figures and images for: A case-control study on association of proteasome subunit beta 8 (PSMB8) and transporter associated with antigen processing 1 (TAP1) polymorphisms and their transcript levels in vitiligo from Gujarat
Source: PLoS One. 2017 Jul 10;12(7):e0180958. doi: 10.1371/journal.pone.0180958 (PMC5507292; doi:10.1371/journal.pone.0180958)

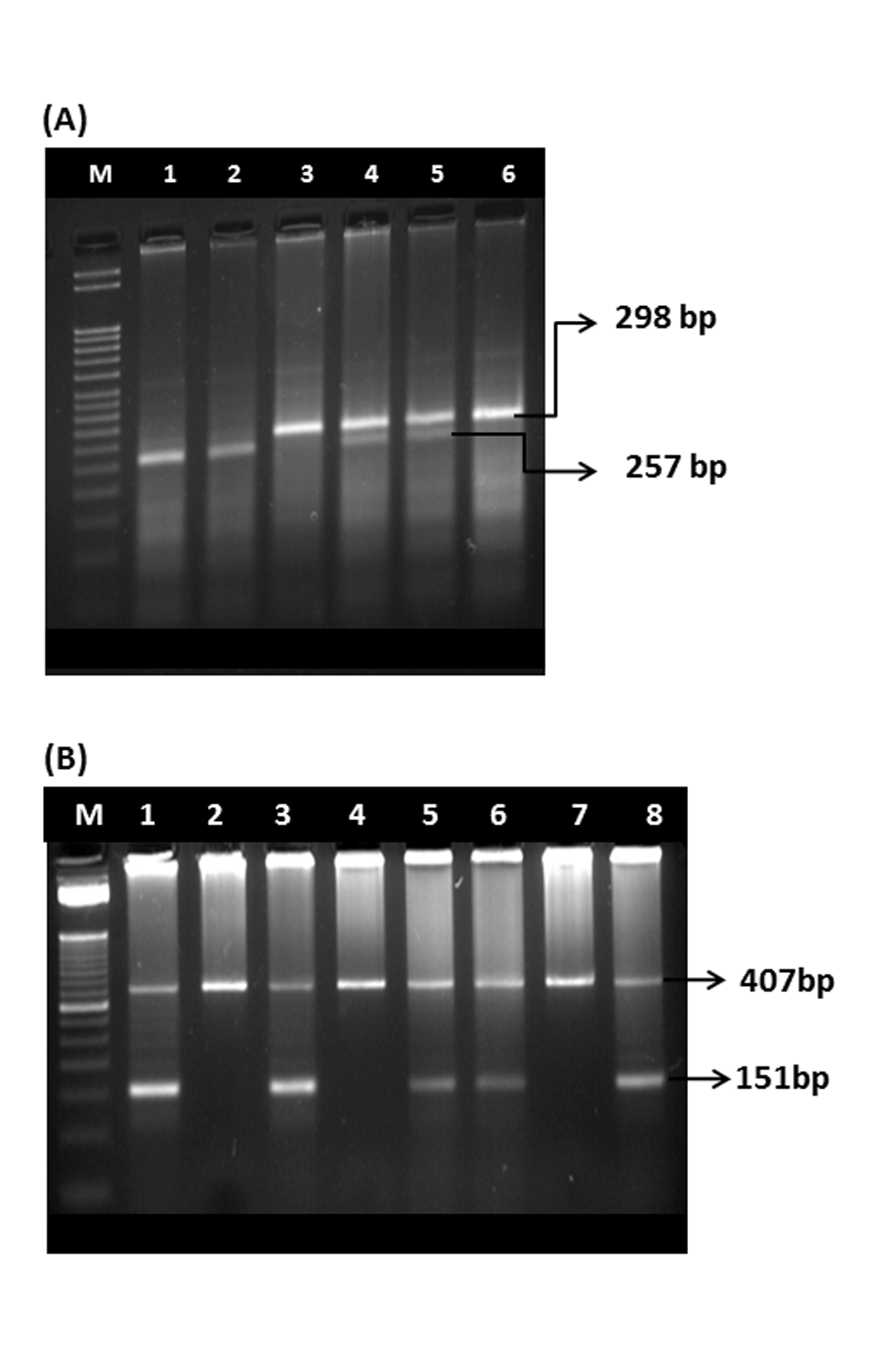

Supplement: S1 Fig — (A) PCR-RFLP analysis of PSMB8 rs2071464 SNP on 3.5% agarose gel: Lane M shows 50bp DNA ladder, lanes: 1 & 2 show homozygous (CC) genotypes; lanes: 3 & 6 show homozygous (TT) genotypes and lanes: 3 & 6 show heterozygous (CT) genotypes. (B) ARMS-PCR analysis of TAP1 rs1135216 SNP on 3.5% agarose gel: Lane M shows 50bp DNA ladder, lanes: 1, 2 & 3, 4 show homozygous (AA) genotypes; lanes: 5, 6 shows heterozygous (AG) genotype and lanes: 7, 8 shows homozygous (GG) genotype. (TIF) [file pone.0180958.s006.tif]

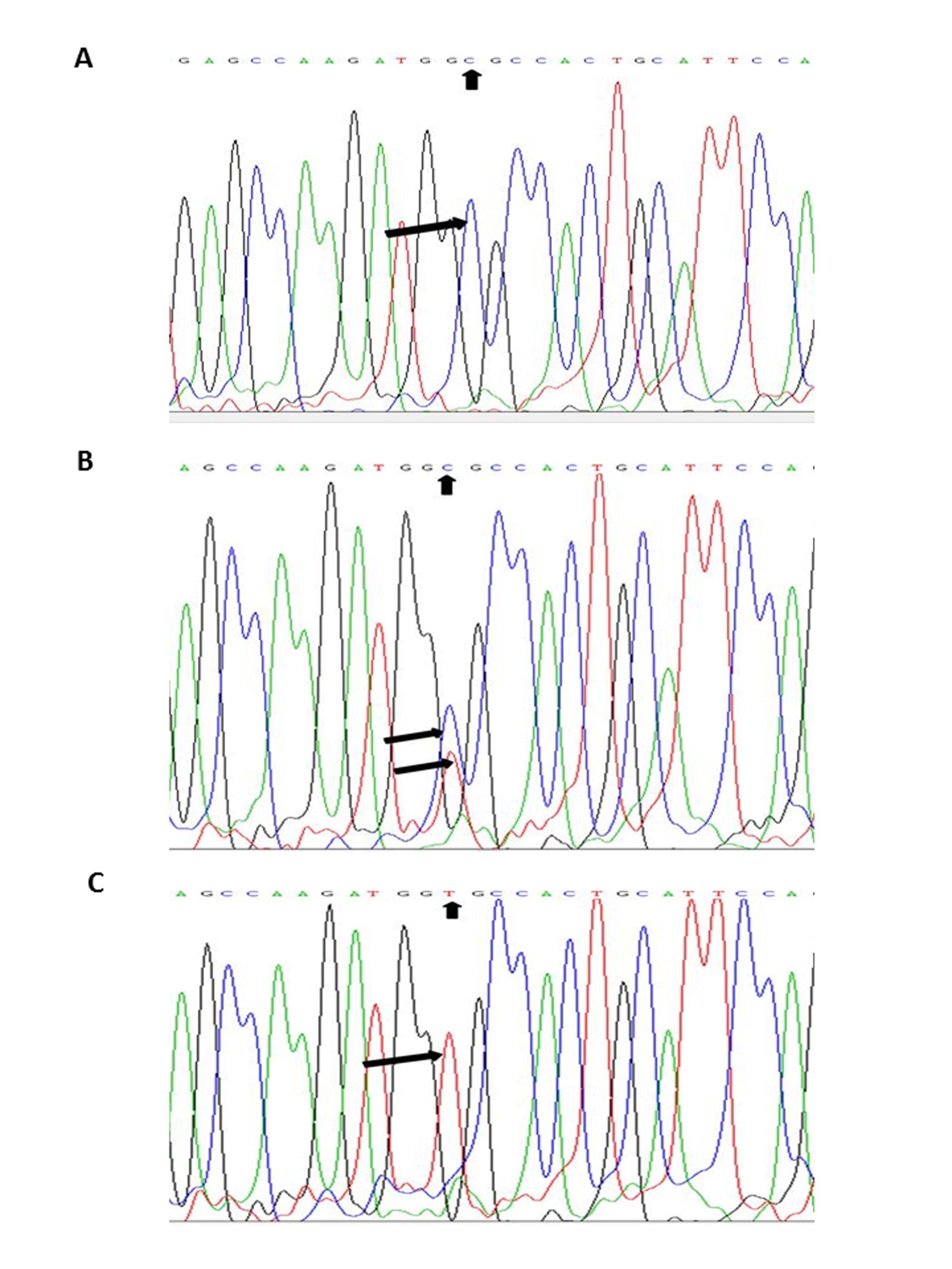

Supplement: S2 Fig — A) PSMB8 rs2071464 CC genotype, B) PSMB8 rs2071464 CT genotype, C) PSMB8 rs2071464 TT genotype. (TIF) [file pone.0180958.s007.tif]

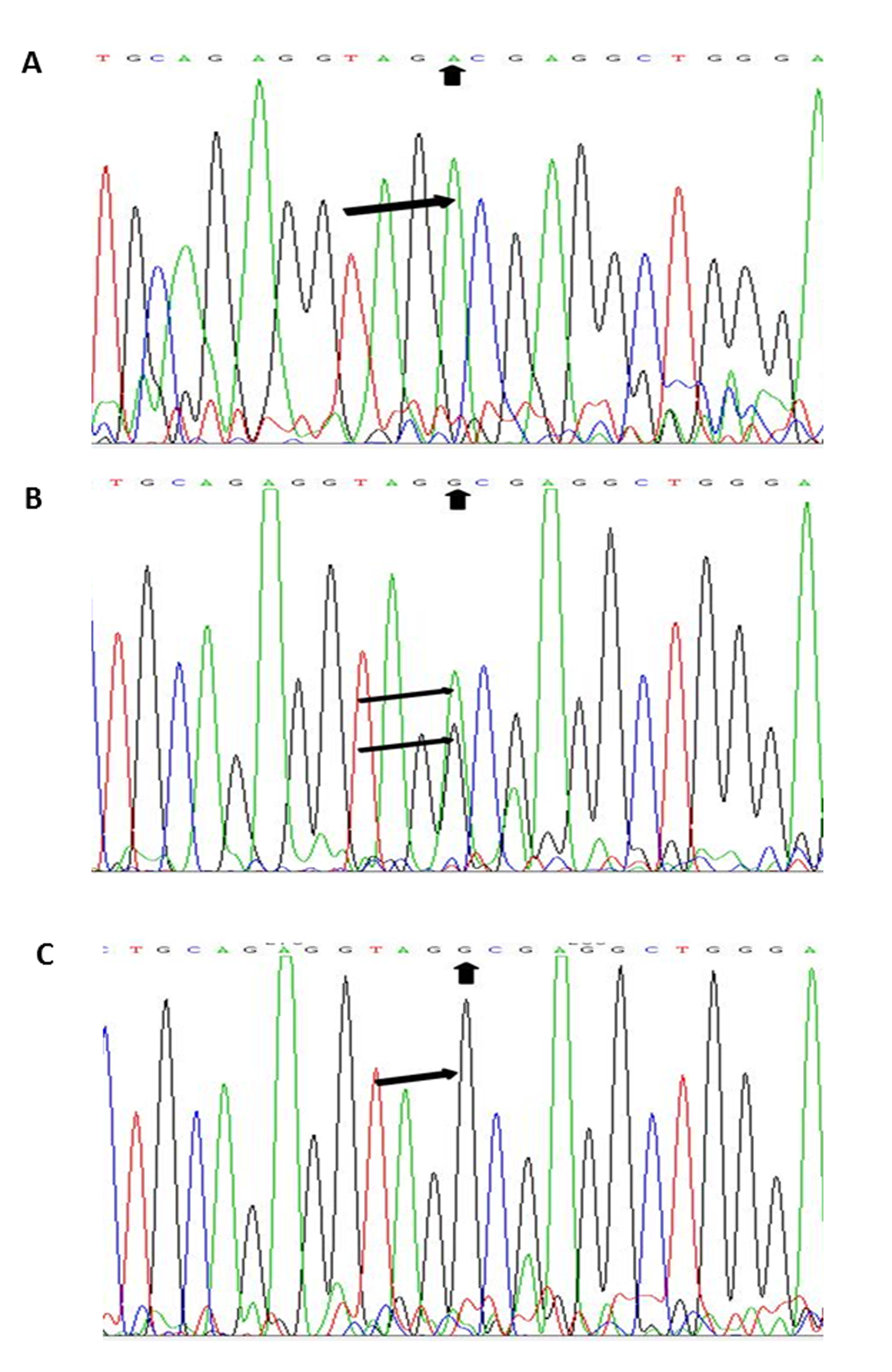

Supplement: S3 Fig — A) TAP1 rs1135216 AA genotype, B) TAP1 rs1135216 AG genotype, C) TAP1 rs1135216 GG genotype. (TIF) [file pone.0180958.s008.tif]
